# Immune checkpoint inhibitors and risk of immune-mediated adverse events: a cohort study comparing extended versus standard interval administration

**DOI:** 10.1007/s10238-024-01301-7

**Published:** 2024-02-22

**Authors:** Amélia Lessard-Roy, Roxanne Marchand, Pierre Lemieux, Mélanie Masse, Alexandre Lacerte, Pierre-Hugues Carmichael, Danielle Laurin

**Affiliations:** 1 Faculté de Pharmacie, Université Laval, Québec, QC Canada; 2 Département de Pharmacie, Hôpital Sainte-Croix, Centre Intégré Universitaire de Santé et de Services Sociaux de la Mauricie-et-du-Centre-du-Québec, Drummondville, Canada; 3 Département de Pharmacie, Centre Hospitalier Affilié Universitaire Régional de Trois-Rivières, Centre Intégré Universitaire de Santé et de Services Sociaux de la Mauricie-et-du-Centre-du-Québec, 1991 Boul. du Carmel, Trois-Rivières, QC G8Z 3R9 Canada; 4 Centre d’excellence sur le Vieillissement de Québec, Centre Intégré Universitaire de Santé et de Services Sociaux de la Capitale Nationale, Québec, Canada

**Keywords:** Immune checkpoint inhibitors, Immunotherapy, Extended interval, Standard interval, Immune-mediated adverse events, Safety

## Abstract

The COVID-19 pandemic precipitated the implementation of extended interval immune checkpoint inhibitors (ICIs) in an effort to limit hospital visits, but few studies have examined their safety. This study aimed to compare in oncology outpatients, immune-mediated adverse events (IMAEs) in terms of total number, incidence, severity, and time to occurrence, based on exposure to standard or extended interval ICIs. A retrospective cohort study was conducted in patients who received at least one dose of an ICI between 2015 and 2021. Data were collected from patient records and pharmacy software. Adjusted logistic, Poisson, and Cox regression models were estimated. A total of 310 patients with a mean age of 67.1 years were included, 130 of whom had the extended interval. No statistically significant differences were observed between the groups. With the standard and extended intervals, the mean total number of IMAE per participant was 1.02 and 1.18, respectively; the incidence of an IMAE was 62% and 64%. Of the 147 IMAE episodes in the standard interval group, 14 (9.5%) were grade 3 or higher, while there were 15 (12.4%) among the 121 IMAE episodes in the extended interval group. Compared with standard interval, the use of extended interval did not increase the risk of having a first IMAE (adjusted hazard ratio 0.92 (95% CI 0.67–1.26)). This study suggests that the administration of an ICI according to extended interval is as safe as the administration according to standard interval in oncology outpatients.

## Introduction

Immunotherapy plays a crucial role in the treatment of various cancers, and immune checkpoint inhibitors (ICIs) have become an important component of the therapeutic arsenal. In the United States, around 40% of cancer patients were eligible for ICI therapy in 2019 [1]. Initially, the standard dosing interval for ICIs was set at 2–3 weeks based on clinical trials and real-world practice. However, the mechanism of action and pharmacokinetics of ICIs support the administration of higher doses at an extended interval of 4–6 weeks, without compromising efficacy and safety [2–5]. Consequently, there has been a growing trend towards adopting the extended interval regimen in clinical practice, which is also recommended by expert panels [3–5].

Pharmacokinetic studies conducted by experts, along with the addition of the extended dosing interval to the Canadian product monograph, have established the use of extended interval nivolumab [2–5]. In a study by Long et al. [2] 61 participants from four ongoing Phase 3 clinical trials who transitioned dosing regimens exhibited a safety profile broadly comparable to the standard interval. Zhao et al. utilized a population pharmacokinetic model and simulated data from 2560 cancer patients, concluding comparable safety for both regimens, although a slightly higher incidence of adverse events was observed in the extended-interval group. In response to the COVID-19 pandemic, this practice has been adopted for pembrolizumab and durvalumab as well, aiming to minimize visits to oncology clinics and reduce the exposure and risk of infection for this vulnerable population, as severe complications could arise from COVID-19 infection [6]. This strategic shift is further substantiated by pharmacokinetic studies. In the context of pembrolizumab, Lala et al. [7] conducted a pharmacokinetic modeling study involving 2993 subjects from five clinical trials. The safety of 400 mg every 6 weeks versus 200 mg every 3 weeks was assessed through simulated "concentration–time" profiles, revealing similar pharmacokinetic exposure for both intervals while ensuring the extended interval remained below the clinically used maximum dose of 10 mg/kg every 2 weeks. For durvalumab, Baverel et al. conducted a population pharmacokinetic modeling study with data from 1409 patients in two studies [8]. Regardless of the four 2 and 4-week regimens covered, the study concluded consistent pharmacokinetic exposure based on the area under the curve of predicted steady-state plasma concentrations. The implementation of less frequent administration intervals not only alleviates the burden on healthcare providers but also conserves valuable human and material resources in the health network, which is especially crucial considering the current state of the health system [5, 9].

Sustaining this practice in the long term offers numerous benefits. By reducing the frequency of administration, the number of patient visits is halved, leading to a positive impact on their overall quality of life. Furthermore, the economic implications for the healthcare system are significant, as various costs associated with the management, preparation, and administration of ICIs can be reduced [5].

Nevertheless, it is important to acknowledge that ICI treatments carry the risk of immune-mediated adverse events (IMAEs) [10]. There is limited evidence available that directly compares the occurrence of IMAEs between standard and extended interval ICI administration. Current practice primarily relies on population pharmacokinetic analyses, indirectly comparing the two administration regimens [2–5, 7, 8, 11, 12]. Only a few clinical studies have evaluated the efficacy and safety of different dosing strategies. The administration of higher doses of monoclonal antibodies at lower frequencies, which theoretically allows for higher peak blood levels, raises safety concerns due to the lack of evidence. As a result, some clinicians are hesitant to adopt the extended therapeutic interval, fearing an increase in the frequency or severity of IMAEs. These concerns are particularly significant in the population with autoimmune diseases, as IMAEs can reactivate their underlying condition when using an ICI [13].

The objective of this study was to compare IMAEs in terms of total number, incidence, severity, and time to first IMAE between patients receiving a standard-interval ICI and those receiving the extended interval. This study also aimed to describe the management of IMAEs and the interventions performed on ICIs for patients experiencing such events. Additionally, the study sought to describe IMAEs according to the therapeutic interval for the subgroup of patients with autoimmune diseases.

## Methods

A retrospective cohort study was conducted using data from the oncology outpatient clinic at the Centre hospitalier affilié universitaire régional (CHAUR) with 432 beds on average and the Hôpital du Centre-de-la-Mauricie (HCM) with 130 beds on average of the Centre intégré universitaire de santé et de services sociaux de la Mauricie-et-du-Centre-du-Québec (CIUSSS MCQ). The CIUSSS MCQ serves a vast territory of 47,000 Km^2^, and corresponds to the sixth most populous socio-health region in the province of Quebec [14]. The CHAUR and the HCM include outpatient clinics in medical oncology, a subspecialty relating to the study, research, diagnosis and medical management of neoplastic pathologies. The interdisciplinary team includes specialist doctors, pharmacists, nurses, psychologists, social workers and nutritionists. The population served by the CIUSSS MCQ included in 2021 about 533,000 inhabitants residing in 121 municipalities and 4 indigenous communities (2% of the region's population). Of these, 18% were under 18 years old, 58% were between 18 and 64 years old and 25% were aged 65 and over. The proportion of people aged 25–64 with a high school diploma was 83%, while those with a university diploma was 15%. The proportion of the population living with low income was 10.5%. Diabetes and high blood pressure (11% and 26%, respectively) were among the most frequent comorbidities of the adult population. Causes of hospitalization include in decreasing order diseases of the circulatory system, respiratory system and digestive system, trauma and poisoning and tumors.

The study involved the same oncology team physicians from both facilities. The study obtained approval from the Research and Ethics Committee (reference number: 2022–608) and received authorization from the Director of Professional Services at CIUSSS MCQ to access patient records.

The study included adult patients (18 years of age or older) who had received at least one dose of nivolumab, pembrolizumab, or durvalumab for cancer treatment between January 1, 2015, and December 31, 2021. To ensure a comprehensive analysis, patients were required to have a minimum follow-up period of 6 months. Patients who received a combination of ICIs (such as nivolumab and ipilimumab) were excluded since this combination is known to increase the risk of unique IMAEs given the addition of ICI and other agents [8]. Patients who received a fixed-dose ICI or weight-base dose exceeding the maximum recommended mg/kg dose specified in the monograph were also excluded, including those participating in other research protocol.

The initial treatment interval with an ICI, either the standard or extended interval, determined the assignment of participants to their respective groups. If a participant changed the treatment interval during treatment, his/her follow-up was terminated on the date of the change, and they were not included in both groups. The standard interval group was defined as follows: nivolumab 3 mg/kg (max 240 mg) every 2 weeks; pembrolizumab 2 mg/kg (max 200 mg) every 3 weeks; durvalumab 10 mg/kg (max 750 mg) every 2 weeks. The extended interval group was defined as follows: nivolumab 6 mg/kg (max 480 mg) every 4 weeks; pembrolizumab 4 mg/kg (max 400 mg) every 6 weeks; durvalumab 20 mg/kg (max 1500 mg) every 4 weeks.

Covariates and potential confounders taken into account included age, biological sex, smoking status (current, former, non-smoker), history of hypothyroidism, liver disease, autoimmune disease, type of cancer (non-small cell lung cancer (NSCLC), melanoma, others), specific molecule received, including dose and dosing interval, as well as characteristics of IMAEs such as affected systems, severity, and the use of corticosteroids or immunosuppressants. Performance status was assessed using the Eastern Cooperative Oncology Group (ECOG) scale, and creatinine clearance was estimated using the Cockcroft-Gault equation [15, 16]. The severity of IMAEs was evaluated according to the Common Terminology Criteria for Adverse Events (CTCAE) version 5.0 [17].

Data were collected via the pharmacy software at the CHAUR and paper medical records at the HCM, which were consulted at the archives service. A data collection tool was developed and pre-tested by ALR and RM. A student from the Pharm.D. program (AL) collected data from HCM patient files under the close supervision of ALR and RM, following training to ensure consistent data collection. All questionnaires were checked by ALR and RM for accuracy and completeness.

### Statistical analysis

Baseline characteristics of groups were compared using the Student's t test for continuous variables and the chi-square test for categorical variables. Incidence data were analyzed with logistic regression, total number of IMAEs per participant with Poisson regression, and time to first IMAE with the log-rank test and Cox regression. All regression models were adjusted for age (continuous), sex, presence of an autoimmune disease (yes/no), cancer type (NSCLC versus all others), and concomitant chemotherapy (yes/no). These covariables were selected a priori based on the published literature [18–20]. A *p* value < 0.05 was considered statistically significant. Analyses were performed using SAS software, version 9.4 (SAS Institute, Inc., Cary, North Carolina).

## Results

Since the baseline characteristics of participants were similar across collection locations, the data were pooled. Out of the initial 430 screened study patients, 120 were excluded for various reasons: participation in another research protocol (n = 46), higher dose exceeding the recommended maximum (n = 37), combination use of ICI (n = 25), failure to meet other eligibility criteria (n = 10), or unavailability of electronic data (n = 2). Thus, the final analysis included 310 participants, with 180 receiving standard-interval ICI and 130 receiving extended-interval ICI. The median duration of follow-up was 8.2 months for participants in the standard interval group and 12.2 months for those in the extended interval group.

Participants’ characteristics were similar between groups (Table 1). In the standard interval group, the average age was 66.9 ± 9.2 years, with 51.7% males. The extended interval group had an average age of 67.3 ± 7.7 years, with 49.2% males. A history of autoimmune disease was observed in only 22 (7.1%) participants, including 14 in the standard interval group. Comparing the two groups, the extended interval group exhibited a significantly lower history of hypothyroidism (13.3 vs. 6.2%) and less concomitant chemotherapy use (9.4 vs. 0.8%). There was a statistically significant difference in the type of cancer between the two groups, with a higher proportion of participants receiving adjuvant treatment for melanoma and fewer receiving treatment for metastatic melanoma in the extended interval group compared to the standard interval group. Furthermore, the standard interval group had a higher utilization of pembrolizumab and lower usage of nivolumab, while the opposite trend was observed in the extended interval group (*p* < 0.001).

**Table 1:** Baseline characteristics of study participants by interval

	Standard interval (n = 180)	Extended interval (n = 130)	*p* value
Age, y	66.9 ± 9.2	67.3 ± 7.7	0.720
Male sex	93 (51.7)	64 (49.2)	0.672
Smoking status			0.979
Non-smoker	30 (16.7)	20 (15.4)	
Former smoker	108 (60.0)	77 (59.2)	
Current smoker	41 (22.8)	29 (22.3)	
Cannabis user	14 (7.8)	8 (6.2)	0.458
Medical history			
Hypothyroidism	24 (13.3)	8 (6.2)	0.040
Hyperthyroidism	1 (0.6)	1 (0.8)	0.817
Autoimmune disease	14 (7.8)	8 (6.2)	0.933
Liver disease	7 (3.9)	8 (6.2)	0.359
Creatinine clearance, ml/min^a^	84.3 ± 31.9	81.7 ± 28.2	0.458
ECOG performance status^b^			0.094
0	38 (21.1)	19 (14.6)	
1	80 (44.4)	68 (52.3)	
2	20 (11.1)	6 (4.6)	
3	2 (1.1)	1 (0.8)	
Type of cancer			0.013
NSCLC			
Adjuvant	12 (6.7)	7 (5.4)	
Metastatic	122 (67.8)	84 (64.6)	
Melanoma			
Adjuvant	9 (5.0)	12 (9.2)	
Metastatic	20 (11.1)	6 (4.6)	
Others	17 (9.4)	21 (16.2)	
Concomitant chemotherapy	17 (9.4)	1 (0.8)	0.001
Reason for discontinuation			0.219
Progression of disease	93 (51.7)	75 (57.7)	
IMAE	26 (14.4)	19 (14.6)	
Completed treatment	25 (14.4)	9 (6.9)	
Molecule			< 0.001
Durvalumab	16 (8.9)	11 (8.5)	
Nivolumab	65 (36.1)	84 (64.6)	
Pembrolizumab	99 (55.0)	35 (26.9)	

Values presented are mean ± standard deviation or n (%)

IMAE, immune mediated adverse event; NSCLC, non-small cell lung cancer

^a^According to Cockcroft-Gault equation

^b^Eastern Cooperative Oncology Group

**Fig. 1:**
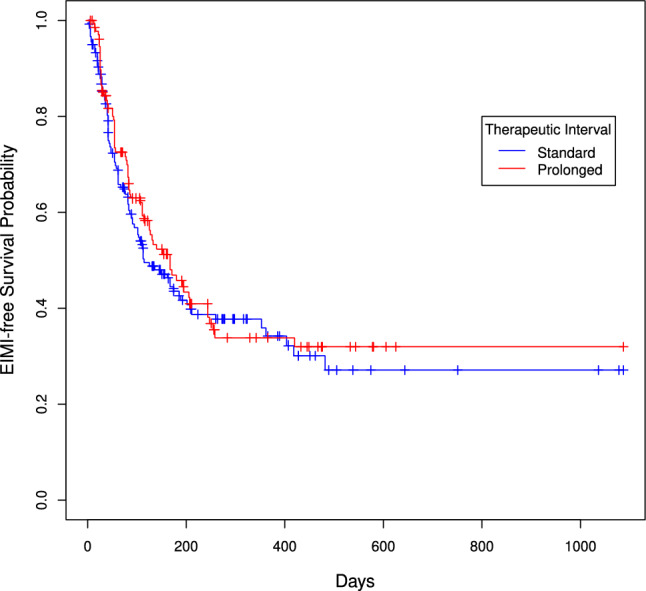
IMAE free survival probability according to treatment interval

No statistically significant differences were found between the two groups of participants regarding the primary outcomes. The average number of IMAE per participant receiving standard-interval ICI was 1.02 (95% confidence interval (CI) 0.73–1.42), while those receiving extended-interval ICI had an average of 1.18 (95% CI 0.81–1.71) (*p* = 0.25). The probability of experiencing at least one IMAE was 62% in the standard interval group and 64% in the extended interval group (adjusted odds ratio of 1.08, 95% CI 0.68–1.72) (Table 2). Among the IMAE episodes, 9.5% (14 out of 147) were grade 3 or higher in the standard interval group, compared to 12.4% (15 out of 121) in the extended interval group. Out of the 180 participants in the standard interval group, 53.9% (n = 97) experienced IMAEs, including 76 with grade 1, 57 with grade 2, and 14 with grade 3 or higher (Table 3). In the extended interval group, out of the 130 participants, 55.4% (n = 72) experienced IMAEs, including 66 with grade 1, 40 with grade 2, and 15 with grade 3 or higher. The median time to the first IMAE was 55 days for the standard interval group and 79 days for the extended interval group (*p* = 0.465). Figure 1 displays the survival curves illustrating time to develop the first IMAE based on the therapeutic interval. The extended interval group did not show a statistically different risk of experiencing a first IMAE compared to the standard interval group (adjusted hazard ratio of 0.92, 95% CI 0.67–1.26).

**Table 2:** Unadjusted and adjusted results according to specific outcomes

	Unadjusted model	Adjusted model^a^
Number of IMAEs per patient^b^	Mean (95% CI)	Mean (95% CI)
Extended interval	0.95 (0.78–1.16)	1.18 (0.81–1.71)
Standard interval	0.82 (0.69–0.98)	1.02 (0.73–1.42)
At least one IMAE^c^	OR (95% CI)	OR (95% CI)
Extended interval	1.06 (0.68–1.67)	1.08 (0.68–1.72)
Standard interval	Reference	Reference
Time to first IMAE^d^	HR (95% CI)	HR (95% CI)
Extended interval	0.90 (0.66–1.22)	0.92 (0.67–1.26)
Standard interval	Reference	Reference

CI, confidence interval; IMAE, immune-mediated adverse events; OR, odds ratio; HR, hazard ratio

^a^Models were adjusted for age, sex, autoimmune disease, cancer type, and concomitant chemotherapy

^b^Results based on Poisson regression modeling. Type 3 test p value for comparison between the extended and the standard intervals is 0.2770 and 0.2470 for unadjusted and adjusted results, respectively

^c^Results based on logistic regression modeling. Type 3 test p value for comparison between the extended and the standard intervals is 0.7942 and 0.7467 for unadjusted and adjusted results, respectively

^d^Results based on Cox proportional hazards regression modeling. Type 3 test p value for comparison between the extended and the standard intervals is 0.4942 and 0.5966 for unadjusted and adjusted results, respectively

**Table 3:** Descriptive characteristics of immune-mediated adverse events by dosing interval (n = 268 events)

	Standard interval			Extended interval		
	Grade 1 (n = 76)	Grade 2 (n = 57)	Grade ≥ 3 (n = 14)	Grade 1 (n = 66)	Grade 2 (n = 40)	Grade ≥ 3 (n = 15)
Cutaneous system	35	12	1	25	9	4
Hepatic system	13	5	2	16	8	3
Gastro-intestinal system	10	12	1	5	10	3
Endocrine system	12	5	1	8	8	2
Pulmonary system	0	11	2	3	1	2
Musculoskeletal system	3	3	2	4	2	1
Neurological system	0	2	2	3	0	0
Renal system	2	2	0	1	1	0
Ocular system	1	2	0	1	1	0
Hematological system	0	2	3	0	0	0
Cardiovascular system	0	1	0	0	0	0
Combined systems						
Support treatment	23	43	13	29	28	13
Corticosteroids	2	34	10	10	26	11
Immunosuppressant	0	4	1	0	1	2

The table includes participants with an IMAE; a participant may have more than one IMAE. Values presented are n

In the standard interval group, participants were more likely to experience a single IMAE rather than multiple IMAEs (36.7% and 17.2%, respectively), whereas in the extended interval group, the proportions were 29.2% and 26.2%, respectively. The IMAEs primarily affected various systems, with the highest occurrence in the cutaneous system (32%), followed by the hepatic system (18%), gastrointestinal system (15%), and endocrine system (13%). Across both groups, IMAEs of grades 1 and 2 were more prevalent (89%) than those of grade ≥ 3 (11%) (Table 3). The number of participants who discontinued treatment due to an IMAE was 26 (14.4%) in the standard interval group compared to 19 (14.6%) in the extended interval group.

Regarding the management of IMAEs based on their severity, corticosteroid therapy was administered in 9.4% of grade 1 IMAEs, 58.1% of grade 2 IMAEs, and 69.2% of grade ≥ 3 IMAEs (Table 4). The average corticosteroid dose for all grades of IMAEs was 0.90 ± 0.44 mg/kg, with an average duration of 101 ± 147 days. Immunosuppressants were not utilized for grade 1 IMAEs, but they were employed in 5.2% of grade 2 IMAEs and 11.1% of grade ≥ 3 IMAEs. Specifically, eight participants received immunosuppressant treatment, with five of them experiencing IMAEs associated with the gastrointestinal system, while the others were related to the hepatic, pulmonary, and hematological systems. In terms of ICI management, nearly 81% of participants who developed grade 1 EIMIs were able to continue their treatment without experiencing any delays in subsequent cycles.

**Table 4:** Characteristics of immune-mediated adverse event management by severity (n = 268 events)

	Grade 1 (n = 142)	Grade 2 (n = 97)	Grade ≥ 3 (n = 29)
Corticosteroid use^a^	13 (9.4)	54 (58.1)	18 (69.2)
Corticosteroid dose^b^			
Without immunosuppressant	0.79 ± 0.56	0.88 ± 0.38	0.81 ± 0.35
With immunosuppressant	n/a	1.26 ± 0.36	1.99 ± 0.02
Duration of corticosteroid^c^			
Without immunosuppressant	163 ± 268	98 ± 141	61 ± 56
With immunosuppressant	n/a	125 ± 154	267 ± 160
Management of ICI			
Continued	90 (63.4)	26 (26.8)	3 (11.1)
Continued with close follow-up	25 (17.6)	1 (1.0)	0 (0)
Suspended^d^	21 (14.8)	50 (51.5)	18 (62.1)
Discontinued^e^	1 (0.7)	10 (10.3)	7 (25.9)
Already discontinued^f^	5 (3.5)	10 (10.3)	1 (3.7)

Values presented are means ± standard deviation or n (%)

ICI, immune checkpoint inhibitor; n/a, not applicable (no data)

^a^Information was missing for 11 patients on corticosteroid use (4 for grade 1; 4 for grade 2; and 3 for grade ≥ 3)

^b^Corticosteroid dose in prednisone equivalent (mg/kg). Information on corticosteroid dose without immunosuppressant included 13 patients for grade 1; 49 for grade 2; and 16 for grade ≥ 3). Information on corticosteroid dose with immunosuppressant included 5 patients for grade 2; and 2 for grade ≥ 3)

^c^Duration of corticosteroid therapy including weaning time (days). Information on duration of corticosteroid without immunosuppressant included 8 patients for grade 1; 43 for grade 2; and 14 for grade ≥ 3). Information on duration of corticosteroid with immunosuppressant included 5 patients for grade 2; and 2 for grade ≥ 3)

^d^ICI suspended for a while to process the IMAE and then resumed

^e^ICI discontinued due to IMAE

^f^ICI treatment terminated for any reason and onset of IMAE after the termination date

At the initiation of treatment, only 22 participants (7.1%) had a pre-existing diagnosis of autoimmune disease. Among the 14 participants with autoimmune disease in the standard interval group, 8 individuals experienced a total of 15 IMAEs. In the extended interval group, out of the eight participants with autoimmune disease, five individuals developed a total of seven IMAEs. Notably, there were three cases of grade 3 IMAEs in the standard interval group, while no grade 4 IMAEs were reported in the autoimmune disease population of either group. Decompensation of the autoimmune disease was observed in 3 out of 14 participants in the standard interval group and in 4 out of 8 participants in the extended interval group.

## Discussion

This study aimed to investigate the occurrence of IMAEs in cancer patients receiving nivolumab, pembrolizumab, or durvalumab under either a standard or extended interval immunotherapy regimen. The findings of this study indicate that the two treatment intervals yielded comparable results. The total number of IMAEs per participant was similar regardless of the dosing interval, as was the incidence and severity of IMAEs. The probability of experiencing at least one IMAE was approximately 60% in both treatment intervals. Furthermore, the observed IMAEs in both groups were predominantly of low to moderate severity. With respect to the time to first IMAE, the results suggest a longer time to onset for the extended interval group.

The findings of this study build upon previous research conducted in pharmacokinetic studies, providing further support for the comparable safety of the two therapeutic regimens. It has been observed that receptor saturation of autoimmunity plays a role in mitigating the exacerbation of IMAEs, despite the higher doses administered in the extended-interval regimen as compared to the standard-interval regimen [5, 21–23]. Moreover, population pharmacokinetic modeling studies, which serves as the basis for current clinical practice, have consistently shown that the extended interval regimen exhibits a safety profile that is largely similar to the standard interval regimen [2, 5, 9–12]. In essence, our study results clinically validate the anticipated outcomes derived from pharmacokinetic and pharmacodynamic studies—specifically, that the administration of checkpoint inhibitors at an extended interval does not induce a higher incidence of IMAEs, nor does it result in a more severe presentation. These findings provide substantial support to the practice of extended administration, challenging the notion that adhering to standard intervals solely due to concerns about IMAEs is warranted. It is important to note that certain circumstances unique to autoimmune diseases may warrant exceptions to this general guideline.

In the context of the COVID-19 pandemic, we acknowledge the existing apprehension regarding the possible unopposed T-cell activation and excessive cytokine release stemming from the intersection of ICI and COVID-19 toxicity, a matter that remains subject to debate [24, 25]. Nevertheless, it is essential to clarify that our study was not designed to substantiate this relationship. Instead, our primary objective is to proactively mitigate the risk of virus exposure and contamination among cancer patients undergoing ICIs. This is achieved by strategically minimizing visits to the hospital-based oncology clinic, representing a preventive approach to enhance patient safety.

Supporting evidence from a recent descriptive study involving 30 participants also documented the presence of mild to moderate IMAEs following modifications to dosing regimens during the pandemic [7]. Furthermore, preliminary results from the ongoing international randomized open-label Phase IIIb/IV CheckMate-384 trial reinforce the comparable safety profile between the two treatment regimens [26]. The study enrolled 329 participants, with 166 receiving nivolumab 480 mg every 4 weeks and 163 receiving nivolumab 240 mg every 2 weeks, and the median follow-up was 9.5 and 10.2 months, respectively. The interim analysis revealed that the occurrence of IMAEs of all grades attributed to treatment at the extended interval or standard interval was observed in 48% versus 61% of participants, respectively. Treatment discontinuation due to adverse events was reported in 6% versus 9% of participants. These results suggest that the utilization of an extended interval does not increase the rate of ICI discontinuation due to adverse events.

It is worth noting that a higher proportion of patients in the extended interval group received nivolumab, while the standard interval group predominantly received pembrolizumab. This discrepancy is not indicative of an indication bias but rather reflects the earlier implementation of extended intervals for nivolumab, spanning over a year. It should also be mentioned that for many indications, pembrolizumab is administered in combination with chemotherapy, which automatically entails the same dosing frequency of every 3 weeks.

Furthermore, the extended interval group had a longer follow-up period of 4 months, indicating that these patients received treatment for a longer duration. This discrepancy in follow-up duration can be attributed to the differing indications for each group, such as adjuvant versus metastatic settings and first-line versus second-line treatments for metastatic disease.

The recent trend in ICI approvals leans towards a fixed-dose regimen, in contrast to older indications that are contingent upon body mass. This dichotomy raises the potential for dosing variability between the standard and extended interval groups. However, it is essential to highlight the implementation of a weight-based dosing strategy (mg/kg) up to the prescribed maximum dose within our center since 2018. Moreover, it is pertinent to note that patients administered a fixed-dose ICI exceeding the stipulated maximum recommended mg/kg dose, as outlined in the monograph, were systematically excluded from the study. Consequently, the probability of significant dosing discrepancies between the study groups impacting the results appears remote. While this exclusion might impact the generalizability of the findings to broader populations, it does not compromise the internal validity of the study's conclusions.

Regarding the time to onset of the first IMAE, it is important to exercise caution in interpreting these results. It is uncertain whether the actual onset of IMAEs is delayed in the extended interval group or if the detection of IMAEs is simply delayed due to the less frequent follow-up visits in this group.

While the onset of late-onset IMAEs can be unpredictable, the vast majority tends to occur within a 4–12 week range [27, 28]. In our retrospective study, we deemed a minimum follow-up time of 6 months as a judicious balance between feasibility and clinical relevance. This duration surpasses the 90-day threshold employed by other studies, enabling a thorough exploration of pertinent factors without sacrificing the practicality of our research [29].

The present study findings reinforce previous literature regarding the prevalence of IMAEs, with skin disorders being the most frequently observed category (32%). This aligns with the reported incidence range in the literature (37–42%) [8]. Moreover, the study revealed a notable occurrence of IMAEs affecting the hepatic, gastrointestinal, and endocrine systems. Hepatic involvement (18%) was generally reported at a lower frequency in the literature (< 5%), while gastrointestinal (15%) and endocrine manifestations (13%) were within the upper range of reported incidences (up to 20% and 5–20%, respectively) [8, 30, 31].

In terms of IMAE management, the average prednisone equivalent dose for corticosteroid therapy (0.90 mg/kg) in this study aligns with results from other studies (0.88 mg/kg) [32]. The dosage of corticosteroid therapy is also consistent with the severity grade of IMAEs, with higher doses administered for grades 3 and 4 compared to grades 1 and 2 [32]. Conversely, a higher dosage of corticosteroid therapy is observed when immunosuppressants are used. As anticipated, the frequency of corticosteroid use increases with the severity of IMAEs.

The response to corticosteroid therapy was generally satisfactory, as evidenced by the infrequent need for additional immunosuppressive therapy. It is worth noting that an inverse relationship was observed between the duration of corticosteroid therapy and the grade of IMAEs, which may seem counterintuitive [32]. However, it is important to consider that out of the two participants who experienced a very severe grade of IMAEs, one passed away shortly thereafter. Consequently, the duration of systemic corticosteroid therapy was shortened in this case.

In the subgroup analysis of participants with autoimmune disease, both the standard and extended interval groups showed a similar probability of IMAE occurrence, approximately 60%, mirroring the findings of the total sample. These results challenge the prevailing literature, which often depicts individuals with autoimmune disease as potentially more susceptible to IMAEs when treated with an ICI [5, 33]. Due to the small number of participants with autoimmune disease (n = 22), it is not possible to compare results by interval received.

To our knowledge, this study includes the largest number of participants specifically addressing the safety of different ICI dosing regimens. The limited number of exclusion criteria as well as the inclusion of all types of cancer that may require the use of an ICI are additional strengths that support the generalizability of the results. While the study predominantly includes a relatively homogeneous white population, there is no evidence to suggest that socio-demographic factors have an impact on the frequency or intensity of IMAEs [18, 30].

However, several limitations should be considered. The retrospective study design relied on information recorded by healthcare professionals, potentially introducing some non-differential misclassification. In addition, the number of IMAEs may be underestimated if a participant sought treatment outside the facilities included in the study. However, it is unlikely that a patient experiencing a significant IMAE requiring medical attention would go unnoticed at subsequent follow-up visits. Monitoring of IMAEs primarily occurred during medical follow-ups and ward-based treatment administration, with participants having the option to contact the oncology team at any time. Participants on the extended-interval treatment had fewer follow-up visits compared to those on the standard interval, potentially explaining the longer time to the occurrence of a first IMAE in the extended-interval group. However, despite these differences in follow-up frequency, there does not appear to be a clinically significant impact, as the total number of IMAEs per participant, the incidence and severity of IMAEs, and the rate of discontinuation of ICI due to IMAEs were comparable between the two groups. Implementing participant censorship for those undergoing transitions between intervals introduces a potential source of non-differential information bias. While the exclusion of individuals experiencing interval changes was a viable option, we contend that the resulting reduction in participant numbers would exert a more substantial influence on the generalizability of the results than the non-differential information bias itself. Furthermore, it is crucial to underscore that the prevalent trend in transitions was from the standard interval to an extended interval. Persistently monitoring these participants would inevitably introduce a differential information bias, potentially exerting a considerable impact on the study results.

## Conclusion

The findings of this study provide reassurance regarding the comparable safety of extended-interval ICI compared to standard-interval ICI in patients who received nivolumab, pembrolizumab, or durvalumab. This study strongly advocates for extended administration, casting doubt on the necessity of strictly adhering to standard intervals based solely on IMAE concerns, with recognition that specific circumstances in autoimmune diseases may warrant exceptions. This is particularly important as current practice trends towards administering ICIs at extended intervals, offering significant benefits for both patients and the healthcare system. In future studies, it would be valuable to investigate whether transitioning from one treatment regimen to another has any impact on treatment tolerance. Understanding the potential effects of such transitions can further inform clinical decision-making and optimize patient care.

## Data Availability

The data and material are available as described in the methods and within this publication. Analyses were performed using SAS software (version 9.4).
